# Disparities in Tumor Mutational Burden, Immunotherapy Use, and Outcomes Based on Genomic Ancestry in Non–Small-Cell Lung Cancer

**DOI:** 10.1200/GO.21.00309

**Published:** 2021-11-09

**Authors:** Otis W. Brawley, Patricia Luhn, Deonna Reese-White, Uzor C. Ogbu, Sriraman Madhavan, Gerren Wilson, Meghan Cox, Altovise Ewing, Christian Hammer, Nicole Richie

**Affiliations:** ^1^ Johns Hopkins University, Baltimore, MD; ^2^ Genentech Inc, South San Francisco, CA; D. R-W. is now with University of Texas at Austin, Austin, TX. U.C.O. is now with Merck & Co Inc, Kenilworth, NJ.

## Abstract

**PURPOSE:**

In patients with advanced non–small-cell lung cancer (aNSCLC), tumor mutational burden (TMB) may vary by genomic ancestry; however, its impact on treatment outcomes is unclear. This retrospective, observational study describes treatment patterns of patients with aNSCLC by genomic ancestry and electronic health record (EHR)-reported race and/or ethnicity and evaluates differences in TMB, cancer immunotherapy (CIT) access, and treatment outcomes across racial and ancestral groups.

**METHODS:**

Patients diagnosed with aNSCLC after January 1, 2011, were selected from a real-world deidentified clinicogenomics database and EHR-derived database; continuously enrolled patients were evaluated. Race and/or ethnicity was recorded using variables from the EHR database; genomic ancestry was classified by single-nucleotide polymorphisms on a next-generation sequencing panel. A threshold of 16 mutations per megabase was used to categorize TMB status.

**RESULTS:**

Of 59,559 patients in the EHR-derived database and 7,548 patients in the clinicogenomics database, 35,016 (58.8%) and 4,392 (58.2%) were continuously enrolled, respectively. CIT use was similar across EHR-reported race groups, ranging from 34.4% to 37.3% for non-Hispanic Asian and non-Hispanic Black patients, respectively. TMB levels varied significantly across ancestry groups (*P* < .001); patients of African ancestry had the highest median TMB (8.75 mutations per megabase; interquartile range, 4.35-14.79). In patients who had received CIT, high TMB was associated with improved overall survival compared with low TMB (20.89 *v* 11.83 months; hazard ratio, 0.60; 95% CI, 0.51 to 0.70) across genomic ancestral groups.

**CONCLUSION:**

These results suggest that equitable access to next-generation sequencing may improve aNSCLC outcome disparities in racially and ancestrally diverse populations.

## INTRODUCTION

Non–small-cell lung cancer (NSCLC) comprises 85%-90% of all primary lung cancer cases, with more than half of all patients presenting with advanced, unresectable disease at diagnosis.^1,2^ The 5-year relative survival rate is 23% in all stages of NSCLC combined.^3^

**CONTEXT**

**Key Objective**
Do racial and ancestral disparities in tumor mutational burden (TMB), cancer immunotherapy (CIT) use, and treatment outcomes exist in patients with non–small-cell lung cancer?
**Knowledge Generated**
Retrospective analysis of data from a large, real-world clinicogenomics database found that reported race was highly correlated with genomic ancestry, and CIT use was similar across groups. However, we found differences in TMB on the basis of genomic ancestry, with patients of African ancestry having significantly higher median TMB scores (8.75 mutations/megabase) than patients of other ancestries. Across all ancestral groups, high TMB was predictive of improved overall survival in patients who received CIT.
**Relevance**
Inequitable access to next-generation sequencing testing has been reported previously; our results suggest that ensuring equal access to testing and CIT across racially and ancestrally diverse populations may improve outcome disparities in patients with advanced non–small-cell lung cancer.

The overall prognosis for NSCLC remains poor, and prognostic factors associated with shorter survival include sex, race, and smoking status.^4-6^ Generally, African American patients have disproportionately higher NSCLC incidence rates and worse outcomes compared with White patients.^7-9^ Multiple factors contribute to racial disparities in the incidence and survival of patients with NSCLC.^10,11^ Frequently, these differences are attributed to social determinants, such as differing smoking habits and access to and quality of care.^12^ Retrospective cohort studies have reported that lower socioeconomic status is an independent prognostic factor for poor survival in patients with early or advanced NSCLC (aNSCLC).^13,14^

Although studies have shown that African American patients are overall less likely to receive treatment, when treatment is controlled for, survival outcomes are similar across racial and ethnic groups.^15,16^ Variations in biologic drivers may also contribute to outcomes; recent studies have reported that the genomic profile of several cancers, including NCSLC, vary by race and ethnicity. This supports the need for greater understanding of the intersection between social determinants of health and biologic drivers in treatment disparities in NSCLC.

The prognosis for patients with NSCLC has improved because of emerging therapies, including cancer immunotherapy (CIT), and increased understanding of disease drivers. Tumor mutational burden (TMB) is a biomarker that may predict response to CIT.^17^ High TMB was predictive of better outcomes in patients with aNSCLC treated with CIT, compared with those who had high TMB but did not receive CIT.^18-20^ As with other genetic biomarkers and targetable mutations, racial differences in TMB have been reported.^21,22^ Although differences in TMB levels have been described in NSCLC, the impact on treatment outcomes by ancestral composition has not been evaluated.

The objectives of this study were to describe the characteristics and treatment patterns of patients with aNSCLC by genomic ancestry and electronic health record (EHR)-reported race and/or ethnicity and to explore potential differences in TMB and disparities in the access to CIT and treatment outcomes across genomic ancestral groups. We also sought to assess the degree of concordance between genomic ancestry and EHR-reported race and/or ethnicity.

## METHODS

### Data Sources

This retrospective, observational study used secondary data evaluating outcomes of patients diagnosed with aNSCLC between January 1, 2011, and March 31, 2020, who were selected from the nationwide (US-based) deidentified Flatiron Health (FH)-Foundation Medicine Inc (FMI)-linked clinicogenomics database (CGDB) and FH EHR-derived database. Patients included in the FH-FMI aNSCLC CGDB had undergone comprehensive genomic profiling (CGP) by FMI at any point. The deidentified data originated from approximately 280 US cancer clinics (≈800 sites of care). Retrospective longitudinal clinical data were derived from EHR data, comprising patient-level structured and unstructured data, curated via technology-enabled abstraction, and linked to genomic data derived from FMI CGP tests in the FH-FMI CGDB by deidentified, deterministic matching.

Genomic alterations were identified via CGP of > 300 cancer-related genes on FMI's next-generation sequencing (NGS)-based FoundationOne panel.^23-25^ To date, more than 400,000 samples have been sequenced from patients across the United States. The majority of patients in the database were from community oncology settings, but relative community or academic proportions may vary depending on study cohort.

### Study Population

Patients who met specific criteria in the aNSCLC FH database and FMI-linked CGDB were included in cohort 1 and cohort 2, respectively. Cohort 3 and cohort 4 comprised patients from cohorts 1 and 2, respectively, who were continuously enrolled in the FH network, defined as no visit gap of > 90 days from date of aNSCLC diagnosis to death or last visit. Cohorts 3 and 4 were used for the main analyses as this allowed for appropriate classification of a patient's line of therapy (LOT; ie, treatment sequence). Detailed inclusion criteria for the four analytic cohorts are described in the Data Supplement.

Patients from the FH database (cohort 1) were categorized into the following groups using the race and ethnicity variables provided: non-Hispanic White, non-Hispanic Black, non-Hispanic Asian, Hispanic or Latino, other non-Hispanic (ethnicity not recorded as Hispanic or Latino and race recorded as Other), or unknown (both race and ethnicity were recorded as null). For the CGDB cohort (cohort 2), a single race and/or ethnicity variable was provided by FH (White, Black, Asian, other, or missing). These variables were used for EHR-reported race comparisons. Derivation of genomic ancestry (European, African, Asian, American, and unknown) has been described previously by FMI.^24^ Briefly, approximately 40,000 single-nucleotide polymorphisms (SNPs) across multiple baitsets (exact number of SNPs may vary by baitset) were used to classify patients into ancestral groups using a principal component analysis with the publicly available 1000 Genomes data as the reference genome. TMB status was categorized as high or low using a threshold of 16 mutations per megabase (mut/Mb). A single, fixed TMB threshold has not yet been defined, and different TMB thresholds have been used in previous studies.^26-28^ Patients with missing ancestry and/or TMB results were excluded from analysis.

### Outcomes

The primary outcome was overall survival (OS). OS was defined as the time from the date of advanced diagnosis until death; however, patients did not enter the risk set until their FMI report date to account for the immortal time bias that exists in the database. As month and year of death were provided, the date of death was imputed to the 15th of the month for all patients with a death date recorded.

### Statistical Analyses

Analysis of variance for continuous variables and χ^2^ analysis for categorical variables were used to summarize the demographic and clinical characteristics for the different cohorts.

For analysis of CIT use, the first LOT that included any CIT (atezolizumab, pembrolizumab, ipilimumab, durvalumab, or nivolumab) was considered the index CIT. The duration of CIT was calculated using time-to-event analysis (Kaplan-Meier method). Two calculations were used: overall CIT duration and CIT duration of a given LOT. Overall CIT duration was determined using the time to last administration regardless of changes in other treatments as long as CIT was given continuously, to allow for treatment with CIT through multiple lines. CIT duration of a given LOT was determined using the date of first administration of CIT after a given start date, until there was a treatment switch, gap in treatment, or death, whichever occurred first.

Event-free survival estimates were generated for cohorts 2 and 4. The association between TMB and OS was estimated using a series of univariate and multivariate Cox proportional hazards models using the advanced diagnosis date as the index date with delayed entry into the cohort at the FMI report date to account for the left-truncated nature of the data set. The assumption of proportional hazards was tested using the Schoenfeld residuals. Multivariate models used in this study are described in the Data Supplement. Analyses were further stratified by genomic ancestry and use of CIT at any point after advanced diagnosis date. Additional subgroup analysis included CIT-treated patients only.

## RESULTS

### Patient Populations

A total of 59,559 patients with aNSCLC were selected from the FH EHR-derived database (cohort 1); 67.2% (n = 40,003) identified as non-Hispanic White, 8.4% (n = 4,977) as non-Hispanic Black, 2.6% (n = 1,523) as non-Hispanic Asian, 3.3% (n = 1,955) as Hispanic, 8.2% (n = 4,869) as non-Hispanic other, and 10.5% (n = 6,232) as unknown. Smoking was less prevalent in non-Hispanic Asian patients (50.2%) compared with > 80% in non-Hispanic White, non-Hispanic Black, and non-Hispanic other populations. The prevalence of stage IV aNSCLC at diagnosis was highest in non-Hispanic Asian patients (70.5%) followed by 69.0%-60.9% in all other ethnic groups. Median duration of follow-up was longest in non-Hispanic Asian patients (431 days) versus 205-313 days in other ethnic groups.

**FIG 1:**
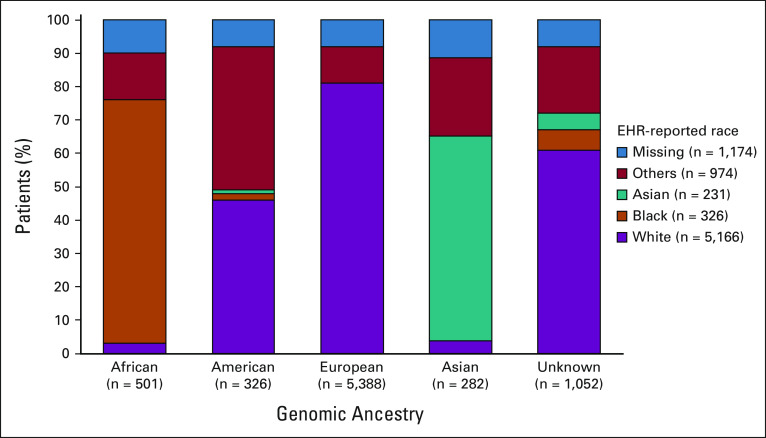
Correlation of EHR-reported race with genomic ancestry (cohort 2). EHR, electronic health record.

A total of 7,548 patients were included from the FH-FMI–linked CGDB (cohort 2). The CGDB provides EHR-reported race information; ethnicity was unavailable. Genomic ancestry distribution was 71.4% (n = 5,387) European, 6.6% (n = 501) African, 4.3% (n = 326) American, 3.7% (n = 282) Asian, and 13.9% (n = 1,052) unknown. A high degree of concordance was seen between EHR-reported race and genomic ancestry (Fig 1).

Of the patients in FH EHR-derived database (cohort 1), 35,016 (58.8%) were continuously enrolled until death or end of study (cohort 3), and from the CGDB (cohort 2), 4,392 (58.2%) were continuously enrolled (cohort 4). The continuous enrollment criteria led to the exclusion of 5,990 and 1,881 patients who received CIT in the EHR-derived database and CGDB, respectively. Continuously enrolled patients in the CGDB (cohort 4) were more likely to be White, male, and stage IV at diagnosis compared with patients who were not continuously enrolled (Table 1).

**TABLE 1:**
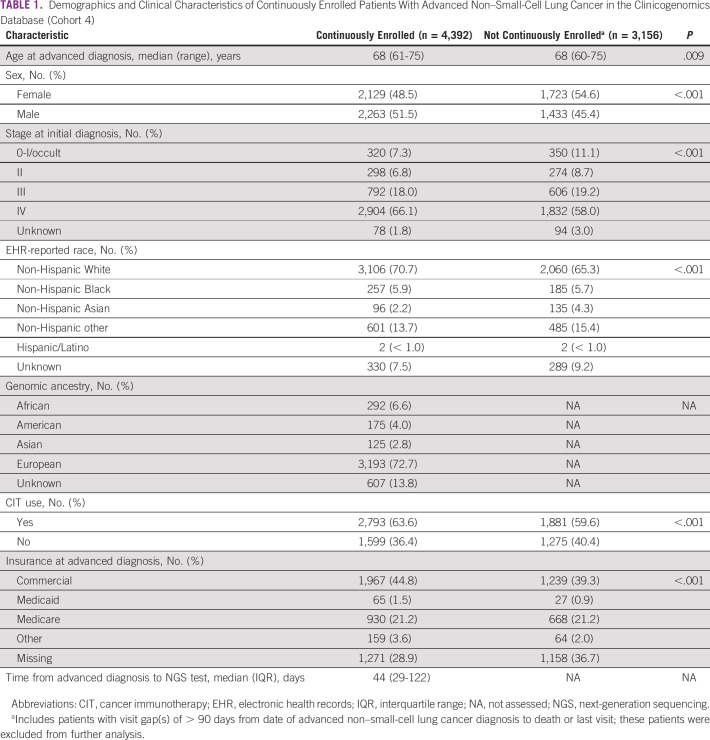
Demographics and Clinical Characteristics of Continuously Enrolled Patients With Advanced Non–Small-Cell Lung Cancer in the Clinicogenomics Database (Cohort 4)

### CIT Use on the Basis of EHR-Reported Race and Genomic Ancestry

In continuously enrolled patients with aNSCLC in the FH EHR-derived database (cohort 3), there was no difference in the prevalence of CIT use on the basis of EHR-reported race. CIT use ranged from 34.4% in non-Hispanic Asian patients to 37.3% in non-Hispanic Black, Hispanic, and non-Hispanic other patients (Table 2). The overall prevalence of CIT use was higher in the CGDB (cohort 4) and was similar across genomic ancestry groups, with 55.2% of Asians having received any CIT, followed by 62.3% of Americans, 62.7% of Europeans, 65.4% of Africans, and 69.7% of patients of unknown ancestry (data not shown). There were no differences on the basis of EHR-reported race or genomic ancestry for the CIT LOT, duration of CIT, or if treatment was monotherapy or combination therapy.

**TABLE 2:**
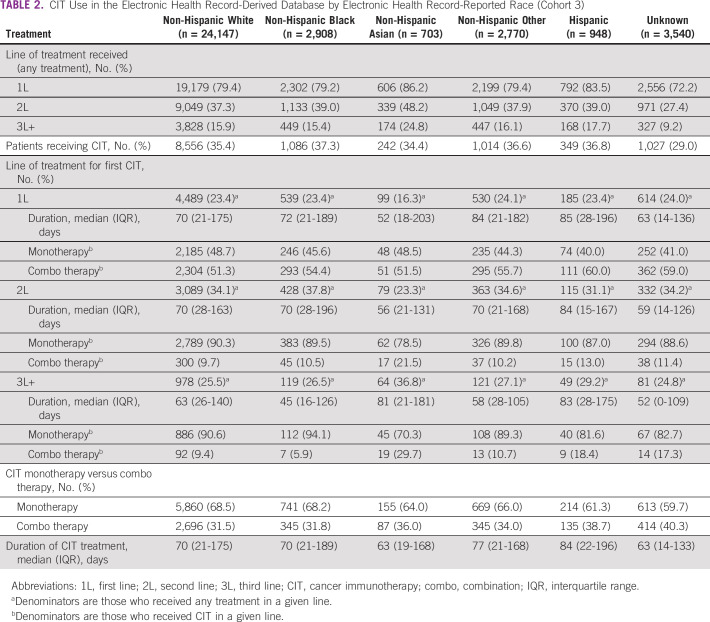
CIT Use in the Electronic Health Record-Derived Database by Electronic Health Record-Reported Race (Cohort 3)

### Association of TMB With Genomic Ancestry and Smoking Status

In continuously enrolled patients with aNSCLC in the CGDB (cohort 4), 13.8% (n = 605) of patients' samples were classified as high TMB and 71.1% (n = 3,121) as low TMB on the basis of the 16-mut/Mb threshold. TMB data were unavailable or missing for 15.2% (n = 666) of patients in cohort 4. TMB status was not associated with any patient characteristics except smoking. The high-TMB group was composed of an increased proportion of patients who smoked (Data Supplement). In addition, there was no difference in the time from advanced diagnosis to FMI test on the basis of TMB status. Across all genomic ancestries, patients with a history of smoking had significantly higher TMB scores when compared with patients with no smoking history (*P* < .001, Data Supplement).

**FIG 2:**
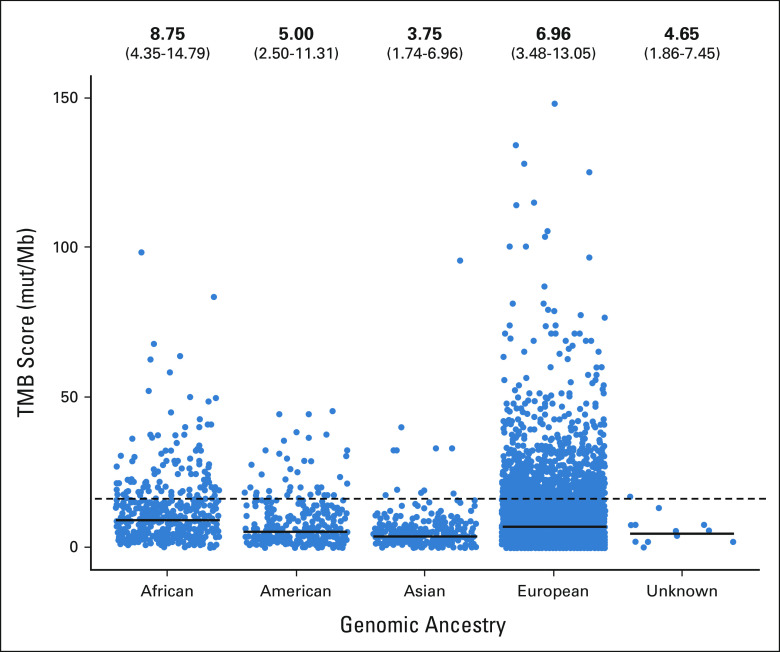
TMB scores by genomic ancestry (cohort 2). Dashed line represents high-TMB threshold (16 mut/Mb); solid line represents median TMB for each ancestral group. IQR, interquartile range; mut/Mb, mutations per megabase; TMB, tumor mutational burden.

Patients of African ancestry had the highest median TMB level (8.75 mut/Mb; interquartile range [IQR], 4.35-14.79), and patients of Asian ancestry had the lowest median TMB level (3.75 mut/Mb; IQR, 1.74-6.96) (Fig 2). High TMB accounted for 19.6% of patients of African ancestry compared with only 3.3% of patients with Asian ancestry (Data Supplement). In patients of European ancestry, median TMB was 6.96 mut/Mb (IQR, 3.48-13.05), and 16.7% were categorized as high TMB. A history of smoking was equally prevalent in patients of African and European ancestry (85%), which was significantly higher than in patients of American (69%) and Asian (45%) ancestry (data not shown). Differences in median TMB scores across genomic ancestral groups were statistically significant (*P* < .001), and among patients with a history of smoking, those of African ancestry had significantly higher TMB scores (Data Supplement).

### Association of TMB Status and CIT With OS

**FIG 3:**
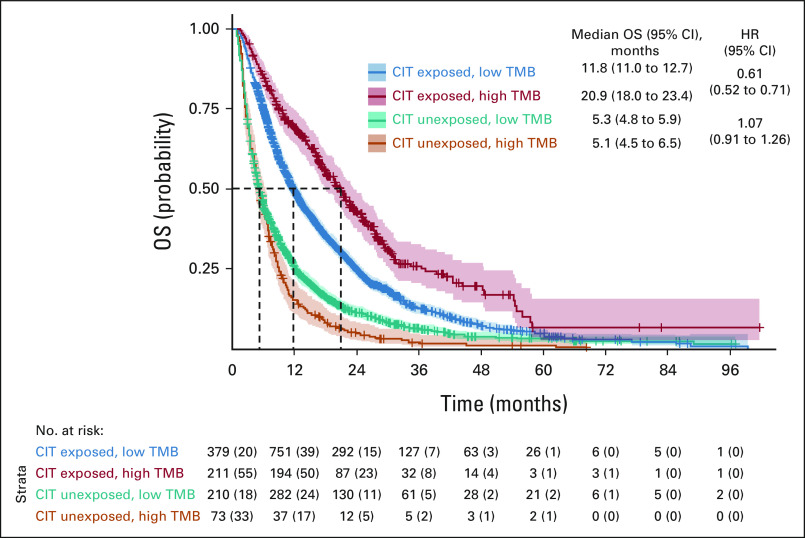
OS (in months) for CIT- and non–CIT-treated patients by TMB status (cohort 4). CIT, cancer immunotherapy; HR, hazard ratio; OS, overall survival; TMB, tumor mutational burden.

In patients who had ever received CIT, high TMB was associated with improved median OS (20.89 months; 95% CI, 17.97 to 23.43) compared with low TMB (11.83 months; 95% CI, 11.04 to 12.68; hazard ratio, 0.61; 95% CI, 0.52 to 0.71; Fig 3). There were no significant differences in median OS between TMB groups in patients who did not receive CIT (hazard ratio, 1.07; 95% CI, 0.91 to 1.26; Fig 3). In CIT-treated patients, high TMB was associated with improved median OS compared with low TMB when patients were stratified by genomic ancestry (Table 3), with similar magnitudes of effect. There was an improvement in OS with CIT use in both high-TMB (20.89 *v* 5.13 months; 95% CI, 4.47 to 6.47 for no CIT) and low-TMB (11.83 *v* 5.29 months; 95% CI, 4.79 to 5.88 for no CIT) groups.

**TABLE 3:**
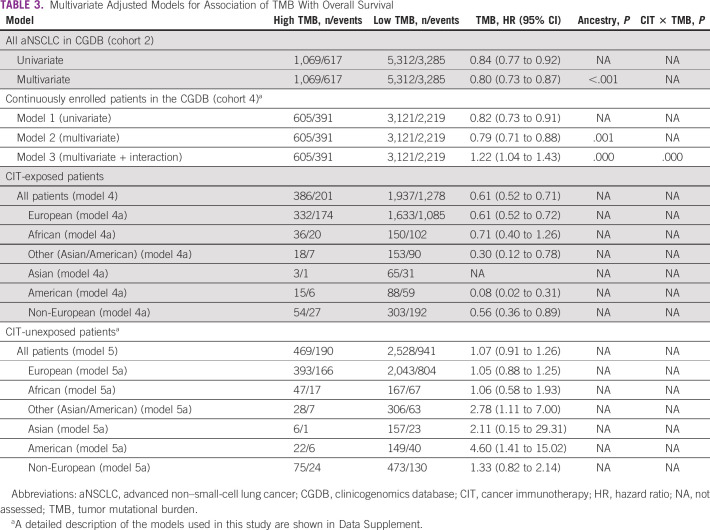
Multivariate Adjusted Models for Association of TMB With Overall Survival

## DISCUSSION

Using a large, comprehensive data set, we demonstrated that genomic ancestry was associated with EHR-reported race. Overall, the results presented here are generally consistent with published literature with respect to distribution of race or ancestry, as well as outcomes in aNSCLC.^9,11,12^

Differences in TMB on the basis of genomic ancestry were observed; patients of African ancestry had significantly higher TMB scores (median, 8.75 mut/Mb), whereas lower TMB scores were observed in patients of Asian ancestry (median, 3.75 mut/Mb). These results are consistent with a previous study in prostate cancer that demonstrated elevated TMB in patients of African ancestry.^29^ When stratified by smoking status, TMB status was also significantly associated with genomic ancestry; patients of African ancestry had the highest median TMB score (10.44 mut/Mb). Across all genomic ancestral groups, patients with a history of smoking had significantly higher TMB scores than those who had never smoked. Previous studies have also demonstrated that smoking is generally associated with higher TMB levels, although the underlying cause is unknown.^30-32^ In this study, median TMB scores across all genomic ancestral groups were lower than the 16-mut/Mb threshold for high TMB. Although a single, fixed TMB threshold has not yet been defined, a TMB threshold of ≥ 10 mut/Mb has been used for patients with NSCLC and established as an indicator for enhanced response to CIT.^26-28^ These results were consistent when a 10-mut/Mb threshold was used; however, because of the reduced response to CIT, the observed differences in OS between high- and low-TMB groups were less pronounced (Data Supplement).

CIT use was not found to vary significantly by EHR-reported race or genomic ancestry, with similar results observed across patient cohorts. However, CIT use was numerically lower for patients of Asian ancestry, who tended to receive CIT in later lines of therapy. This may be attributed to the higher prevalence of *EGFR* mutations in this ancestral group, which renders them suitable candidates for first-line treatment with tyrosine kinase inhibitors rather than CIT.^33^ Treatment with CIT improved OS regardless of TMB status. For patients treated with CIT, TMB status was predictive of improved OS across all genomic ancestral groups with a longer OS observed for high-TMB versus low-TMB groups. However, for patients who did not receive CIT, OS was similar between TMB groups. Additionally, OS was shorter in non–CIT-treated patients than has been previously reported, which may suggest that FMI-tested patients had a poorer prognosis compared with the broader NSCLC population.^34-36^ The median time from advanced diagnosis to FMI NGS testing for continuously enrolled patients in the CGDB ranged from 29 to 122 days, suggesting that some patients may undergo NGS testing later in the course of treatment. Patients who had undergone NGS testing may have more complex or advanced cancer, which may be reflected by the shorter OS observed in this study. It is likely that this discrepancy in OS will change over time as NGS testing becomes more widely incorporated into routine clinical care in the first-line setting.^37^

A limitation of this study is the lack of racial and ancestral diversity in the study population. Approximately 70% of patients identified as non-Hispanic White (cohort 1) or were of European ancestry (cohort 2). In addition, EHR-reported race was highly correlated with genomic ancestry (as defined by FMI). However, capture of EHR-reported race (self or physician reported) was limited to a few predefined categories, whereas genomic ancestry was on the basis of a small number of SNPs; neither method accounts for the influence of genetic admixture. The limited race and ethnicity designations in this real-world database may have increased the potential for missing data. Similarly, the requirement of NGS/TMB testing in the CGDB may introduce selection bias for those with access to, and under the care of, physicians with distinct practice patterns; thus, patients may not be fully representative of the overall aNSCLC population. Engagement with physicians in the FH network may indicate a generally high level of health care access and interaction with the health care network, which may affect the interpretation of any racial disparities. Since all participants had access to NGS/TMB testing and CIT, any observed differences in health outcomes would likely be attributable to biologic factors rather than inequitable access to care. Additionally, because of the current lack of available data, other broader social determinants were not assessed in this analysis.

Overall, no obvious disparities in the use of emerging treatments (CIT) on the basis of EHR-reported race or genomic ancestry were observed. However, there were statistical differences in the prevalence of emerging biomarkers (TMB) by genomic ancestry, with higher TMB in patients of African ancestry despite a similar prevalence of patients with a history of smoking. Although race is a sociocultural categorization, the racial differences in TMB remain poorly understood. Racial differences in TMB status may be influenced by different patterns of tobacco consumption, including the type of tobacco consumed. For example, more than 80% of non-Hispanic Black cigarette smokers in the United States use menthol cigarettes compared with approximately 25% of non-Hispanic White smokers.^38,39^ Menthol cigarettes have been shown to produce positive sensory effects, and evidence suggests that their use is associated with an increased risk of nicotine addiction and long-term daily cigarette use.^40^

Since clinical and demographic characteristics, except smoking, were not associated with TMB status, it appears that high TMB is not associated with more severe disease characteristics. High TMB was predictive of improved OS in patients from different ancestral groups (European, African, and Asian/American ancestries) who were treated with CIT. Given that previous studies using real-world data have reported racial disparities in access to NGS/TMB testing,^41^ the results presented here suggest that equal access to testing and CIT results in improved outcomes in patients with aNSCLC, regardless of ancestral background. These results further underscore the need to ensure equitable access to NGS testing and CIT across racially and ancestrally diverse populations to address and improve disparities in NSCLC outcomes.

## DATA SHARING STATEMENT

The data that support the findings of this study have been originated by Flatiron Health Inc and Foundation Medicine Inc. These deidentified data may be made available upon request and are subject to a license agreement with Flatiron Health and Foundation Medicine; interested researchers should contact cgdb-fmi@flatiron.com to determine licensing terms.
